# Increased microRNA-93-5p inhibits osteogenic differentiation by targeting bone morphogenetic protein-2

**DOI:** 10.1371/journal.pone.0182678

**Published:** 2017-08-10

**Authors:** Ying Zhang, Qiu-Shi Wei, Wei-Bin Ding, Lei-Lei Zhang, Hui-Chao Wang, Ying-Jie Zhu, Wei He, Yu-Na Chai, You-Wen Liu

**Affiliations:** 1 Medical Centre of Hip, Luoyang Orthopaedic-Traumatological Hospital (Orthopaedic Hospital of Henan Province), Luoyang, China; 2 The First Affiliated Hospital, Guangzhou University of Chinese Medicine, Guangzhou, China; 3 Guangzhou Ginkgo Biotechnology Co., LTD., Guangzhou, China; 4 The First Affiliated Hospital of Zhengzhou University, Zhengzhou, China; Kyungpook National University School of Medicine, REPUBLIC OF KOREA

## Abstract

**Background and purpose:**

Trauma-induced osteonecrosis of the femoral head (TIONFH) is a major complication of femoral neck fractures. Degeneration and necrosis of subchondral bone can cause collapse, which results in hip joint dysfunction in patients. The destruction of bone metabolism homeostasis is an important factor for osteonecrosis. MicroRNAs (miRNAs) have an important role in regulating osteogenic differentiation, but the mechanisms underlying abnormal bone metabolism of TIONFH are poorly understood. In this study, we screened specific miRNAs in TIONFH by microarray and further explored the mechanism of osteogenic differentiation.

**Design:**

Blood samples from patients with TIONFH and patients without necrosis after trauma were compared by microarray, and bone collapse of necrotic bone tissue was evaluated by micro-CT and immunohistochemistry. To confirm the relationship between miRNA and osteogenic differentiation, we conducted cell culture experiments. We found that many miRNAs were significantly different, including miR-93-5p; the increase in this miRNA was verified by Q-PCR. Comparison of the tissue samples showed that miR-93-5p expression increased, and alkaline phosphatase (ALP) and osteopontin (OPN) levels decreased, suggesting miR-93-5p may be involved in osteogenic differentiation. Further bioinformatics analysis indicated that miR-93-5p can target bone morphogenetic protein 2 (BMP-2). A luciferase gene reporter assay was performed to confirm these findings. By simulating and/or inhibiting miR-93-5p expression in human bone marrow mesenchymal stem cells, we confirmed that osteogenic differentiation-related indictors, including BMP-2, Osterix, Runt-related transcription factor, ALP and OPN, were decreased by miR-93-5p.

**Conclusion:**

Our study showed that increased miR-93-5p in TIONFH patients inhibited osteogenic differentiation, which may be associated with BMP-2 reduction. Therefore, miR-93-5p may be a potential target for prevention of TIONFH.

## Introduction

TIONFH is an important type of femoral head necrosis caused by femoral neck fractures, hip dislocation and other hip trauma. Patients who develop osteonecrosis eventually suffer bone collapse, and many patients require hip replacement surgery. The facilitation of fracture healing and the preservation of the femoral head for prevention or delay of total hip arthroplasty to avoid osteonecrosis are currently recognised as the goals of TIONFH treatment. Bone metabolism homeostasis is pivotal for fracture healing. The dynamic balance of bone resorption and bone formation primarily involves osteoclasts and osteoblasts. Promoting osteogenic differentiation is an important strategy to enhance bone repair.

MicroRNAs (miRNAs), a class of small, conserved non-coding RNA molecules (averaging 19–22 nucleotides), have important regulatory functions in osteogenic differentiation, including adipogenesis, osteogenesis and cartilage development [1,2,3]. Osteogenic cells derived from bone marrow mesenchymal stem cells (BMSCs) show a relative decrease in number during femoral head necrosis, and this change in activity directly contributes to osteonecrosis.

Human BMSCs (hBMSCs), which have the capacity of self-renewal and differentiation, can differentiate to form a variety of tissues, including bone, cartilage, adipose and endothelium [4,5], which is useful in tissue regeneration. These properties suggest that hBMSCs are potential treatments for various diseases and are critical in regulating bone differentiation. Endogenous miRNAs play a regulatory role in hBMSC osteogenic differentiation. Therefore, we selected hBMSCs for in vitro experiments.

Bone morphogenetic protein (BMP), an endogenous mediator, is necessary for fracture repair [6]. It belongs to the acidic glycoprotein family, is primarily synthesised and secreted from osteoblasts and is widely present in the bone matrix. There are currently more than 20 members in the BMP family, a class of relatively strong osteogenic factors, which are capable of stimulating bone mesenchymal progenitor cells to differentiate into mature osteoblasts. BMP-2 is the most important extracellular signalling molecule, promoting bone formation and inducing bone cell differentiation [7,8], and regulates the gene expression of a variety of transcription factors, such as Osterix and Runx-2, critical osteogenic transcription factors. Runx-2 is expressed in premature osteoblasts, osteoblasts, premature chondrocytes and early hypertrophic chondrocytes. BMP-2 induces Runx-2 expression at both the transcriptional and posttranscriptional levels [9]. Osterix is expressed in all developmental osteoblasts and osteocytes and is positively regulated by BMP-2 during osteogenic differentiation. Loss of BMP-2 results in severely impaired osteogenesis [10]. However, the role of BMP-2 in osteogenic differentiation has yet to be confirmed, and its complementary miRNA as well as the related mechanism remains to be further elucidated.

In this study, we aimed to identify specific miRNAs involved in TIONFH and the mechanism of osteogenic differentiation to provide a foundation for diagnosing and treating TIONFH.

## Materials and methods

### Clinical sample data

The inclusion and exclusion criteria of the samples were as follows: from January to April in 2015, patients with femoral neck fractures in the Henan Orthopaedic Hospital Hip Disease Treatment Study Centre were treated by manual reduction and percutaneous cannulated screw fixation (without gender limitations). Among these patients, 10 met the diagnostic criteria of ONFH (osteonecrosis of the femoral head) [11], and another 10 patients lacked ONFH after treatment. None of the patients had received drug treatment in the last six months, and they lacked other joint diseases, such as gout, rheumatoid arthritis and others. In addition, patients with any systemic inflammation, autoimmune disease, or chronic malignant disease were not included in this study.

All patients underwent evaluation for the following: age, sex, body mass index (BMI), pulse, respiration, body temperature, blood pressure, Harris hip score [12], visual analogue score (VAS score) [13], bilateral hips joint X-ray of orthotopic and frog-leg position, and bilateral hip MRI examination. Bone tissues of the TIONFH patients were evaluated with micro-CT and histological examination [haematoxylin-eosin (HE) staining].

The Harris score was used as hip function scoring system, which includes Pain、Limp、Support、Distance Walked、Sitting、Enter public transportation、Stairs、Put on Shoes and Socks、Absence of Deformity (All yes = 4; Less than 4 = 0)、Range of Motion (indicates normal) and Range of Motion Scale.

VAS score was used as pain scoring system. Draw a 10 cm line on the paper with number 0–10, which indicates varying degrees of pain. Let the patient mark the line according to his feeling, indicating the extent of the pain.

The experimental programme was fully in accordance with the relevant provisions of the 1964 Helsinki Declaration. This statement was approved by the Henan Provincial Hospital orthopaedic clinical trials ethics committee of Luoyang Orthopaedic Hospital. The study team obtained the written consent from each clinical subject and then implemented the experimental programme.

### Screening of differentially expressed genes

Elbow venous blood samples were collected from subjects under fasting conditions and stored at -80°C. Samples were stored until RNA extraction to avoid freezing and thawing. Blood samples were used for microarray detection, and miRNA differences were identified.

### Immunohistochemistry detection

Normal bone tissue and necrotic bone tissue from five osteonecrosis patients were assessed. Immunohistochemistry was used to detect protein expression of BMP-2, ALP and OPN. The femurs were dissected and fixed in 4% paraformaldehyde for 48 h, followed by decalcification in 8% EDTA (pH 7.4) at 4°C. The decalcified specimens were processed for paraffin embedding, and serial 5-mm sections were prepared. The anti-BMP-2 mAb (Santa Cruz) was used at a dilution of 1:300. ALP (Abcam) and OPN (Santa Cruz), antibodies were diluted 1:200. Mouse IgG, at the same concentrations as the corresponding primary antibodies, was used to replace the primary antibodies in the negative control experiments. All immunohistochemistry experiments were performed using the DAB kit according to the manufacturer’s instructions, and images were obtained with an Olympus cx31 system software.

### Target gene prediction

Online software (http://www.microrna.org/microrna/home.do) was used to predict potential target genes of miR-93-5p.

### Cell isolation, culture and identification

The hBMSCs used in this study were isolated from the bone marrow of donors with trauma who had provided informed consent. These hBMSCs were harvested and cultured as previously described [14,15]. hBMSCs were isolated by Ficoll density-gradient centrifugation [16]. A PBS mixture was allowed to stand for 10 min, and the supernatant was slowly transferred to a 15 ml centrifuge tube containing an equal amount of Ficoll-Paque (GE Healthcare, USA). After 35 min of centrifugation (2,000 rpm), mononuclear cells were collected from the interface (the cloud-like cell layer) and resuspended in 8 ml culture medium. Cell lines were cultured in Dulbecco’s modified Eagle’s medium supplemented with 10% heat-inactivated foetal bovine serum, 100 units/ml penicillin, and 100 mg/ml streptomycin in 5% CO_2_ in a humidified incubator at 37°C. All experiments using hBMSCs were performed at passages 2–5. For osteogenic differentiation, cells were cultured using osteogenic induction solution containing 0.1 μmol/L dexamethasone, 50 mg/L ascorbic acid, and 10 mmol/L β-glycerophosphate [17].

hBMSCs were analysed for the expression of CD29, CD34, CD44, CD45, CD29, CD34, CD44, and CD45 by flow cytometry. Approximately 5.0×10^4^ second-passage hBMSCs were stained with 20 μl FITC-conjugated antibody (BD, USA) for 30 min at 4°C. IgG1 was used as a control. Cells were analysed for fluorescence within 6 h using BD Accuri C6 Flow Cytometry.

### Oligonucleotides and plasmids

The miR-93-5p mimic/inhibitor, negative control mimics (NC) and primers were obtained from RiboBio Biotechnology (Guangzhou, China). A 580 bp region of BMP-2 3' untranslated region (UTR), containing the potential miR-93-5p binding site cloned into the luciferase assay plasmid PSICHECK2.0, was obtained from Ginbio (Guangzhou, China) to demonstrate a direct interaction between miR-93-5p and BMP-2. All primers were synthesised by Sangon Biotech (Shanghai, China) (Table 1).

**Table 1:** Primers used for amplification.

Gene	Forward (5'-3')	Reverse (5'-3')
BMP-2	GGTGGAATGACTGGATTGT	GAGATAGCACTGAGTTCTGT
Runx-2	CAGCACTCCATATCTCTACTAT	CTTCCATCAGCGTCAACA
Osterix	CAGGCTATGCTAATGATTACC	GGCAGACAGTCAGAAGAG
ALP	AGATGACTACAGCCAAGGT	CTCCACGAAGAGGAAGAAG
OPN	ATGGCTTTCATTGGAGTTGCTTG	TGGTTTGCCTTTGCCTGTTCG
β-actin	ATGCTGCTTACATGTCTCGAT	AGCAGAGAATGGAAAGTCAAA

### Luciferase reporter assays

To construct a luciferase reporter for wild-type (WT) BMP-2 3'UTR, we synthesised this UTR sequence and sub-cloned it into the NotI and XhoI sites in the apsiCHECK-2 vector (Promega). A mutant BMP-2 3'UTR was generated by site-directed mutagenesis using Phusion^™^ High-Fidelity DNA Polymerase (Thermo Scientific) according to the manufacturer’s protocol. All constructs were confirmed by sequence analysis. For transfection, 5 × 10^4^ 293T cells were plated in complete medium on a 24-well plate. The next day, the cells were transfected with 50 nM of miR-93-5p mimics or NC mimics and inhibitor using Lipo3000 (Thermo Scientific) according to the manufacturer’s protocol. Three groups of cells (miR-93-5p NC group, miR-93-5p mimic group and miR-93-5p inhibitor group) were harvested 72 h after transfection in cell lysis buffer and then assayed for luciferase activity using the Dual-Luciferase Reporter Assay System (Beyotime, Shanghai) and a luminometer according to the manufacturer’s protocol. Transfection of each construct was performed in triplicate for each assay, and the ratios of Renilla luciferase activity to firefly luciferase activity were averaged for each experiment. For protocol see: dx.doi.org/10.17504/protocols.io.irbcd2n.

### MTT assay for cell proliferation

For the MTT assay, 5×10^3^ cells per well were seeded with complete growth medium on a 96-well plate. The cells were divided into three groups: miR-93-5p NC group, miR-93-5p mimic group and miR-93-5p inhibitor group. Treated cells were assessed for 1 to 3 days via the previously described MTT assay. The data were measured using a microtiter plate reader with a 570 nm filter (Thermo Scientific).

### Q-PCR analysis

#### Q-PCR analysis 1: miR-93-5p

Q-PCR for miR-93-5p was performed according to standard protocols using a Bio-Rad CFX96 Touch^™^ Deep Well Q-PCR Detection System. The expression of miR-93-5p was evaluated using a mirVana^™^ qRT-PCR miRNA Detection Kit (RiboBio Biotechnology, Guangzhou). The primers were designed and synthesised by RiboBio Biotechnology (Guangzhou). U6 was used as an internal control.

Total RNA was isolated from the four groups of cells (as above) after 0, 3, and 7 days in differentiation medium with TRIzol (TaKaRa, Dalian, China), and cDNA was synthesised using a special miR-93-5p primer. The quantity and quality of isolated RNA was determined using a 2000 Spectrophotometer (Thermo Scientific).

#### Q-PCR analysis 2: BMP-2, OPN, Runx-2, ALP and Osterix

After 7 days in differentiation medium with osteogenic induction, two cell groups (miR-93-5p NC, and miR-93-5p mimic groups) were harvested, and RNA was reverse-transcribed into cDNA using oligT primers and M-MLV reverse transcriptase (TaKaRa, Dalian, China). For Q-PCR of transcripts, cDNA was mixed with Power SYBR Green PCR Mastermix (Applied Biosystems, Foster City, CA) and analysed on a Bio-Rad Real-Time PCR system. The primers are shown in the Supplementary materials and include primers for BMP-2, OPN, Runx-2, ALP and Osterix. β-actin was used as an internal control (Table 1). Quantification of the fold change in gene expression was determined with the ΔΔCt method.

### Western blotting analysis of BMP-2, Runx-2, OPN, and ALP

After 7 days in differentiation medium with osteogenic induction, two cell groups (miR-93-5p NC group and miR-93-5p mimic group) were seeded on 60 mm plastic dishes (WHB) and cultured for 7 days in osteogenic differentiation medium. Total protein was isolated using RIPA buffer. Proteins were separated by 12% SDS-PAGE and transferred to a PVDF membrane for 1.5 h at 4°C. Membranes were blocked with 5% milk in TBST for 2 h at room temperature and incubated with primary antibodies against BMP-2 (1:200, Santa Cruz, USA), OPN (1:200, Santa Cruz, USA), Runx-2 (1:200; Santa Cruz, USA), ALP (1:100, Boster, China), Osterix (1:200, Santa Cruz, USA), or GAPDH (1:3000, Tianjin Sungene, China) at 4°C overnight. Membranes were incubated with HRP-conjugated secondary antibody (1:2000, Boster, China) for 1 h at room temperature, followed by scanning with X-ray film. The integrated intensity for each detected band was then determined with ImageJ, v.1.46.

### Alkaline phosphate (ALP) assay

Three cell groups (miR-93-5p NC and miR-93-5p mimic groups) were seeded in 24-well plates (Costar) for the ALP activity assay. Cells were harvested and resuspended in 250 μl culture supernatants, which was followed by cell rupturing with an ultrasound breaker. After centrifugation, the ALP activities in the cell supernatants were quantified using an ALP Detection Kit (Nanjing Jiancheng Biotech Institute, China) and a spectrophotometer at a wave length of 520 nm. Each value was normalised to the protein concentration.

### Alizarin red (AR) staining

Two cell groups (miR-93-5p NC and miR-93-5p mimic groups) were seeded on 96-well plastic dishes (Costar) to examine mineralisation. Cells were cultured in differentiation medium for 21 days and then washed twice with PBS and fixed with 4% paraformaldehyde at room temperature for 10 min. The dishes were then washed three times with distilled water and incubated with 0.1% AR (Sigma, USA) at 37°C for 30 min. Cells were thoroughly washed with distilled water, and images were captured using a scanner.

### Statistical analysis

SPSS 19.0 statistical software (SPSS Inc., Chicago, IL) was used for statistical analysis. All data are presented as the mean ± standard deviation. Data between the two groups were analysed using t tests, and multiple comparisons between groups were assessed by one-way ANOVA, assuming double-sided independent variance. P values < 0.05 were considered statistically significa.

## Results

### Clinical data

**Fig 1:**
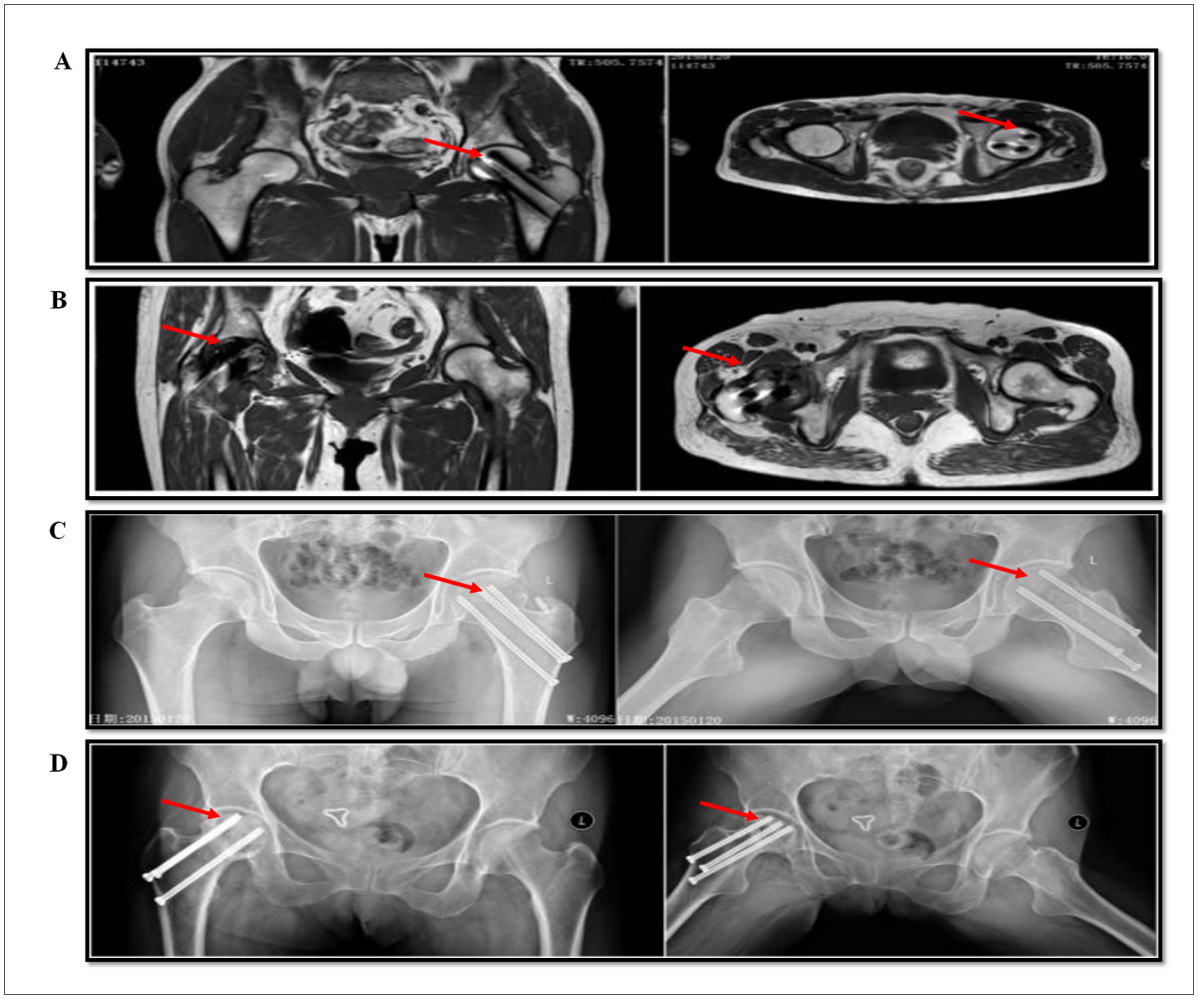
MRI and X-ray examination of TIONFH patients and the patients without necrosis. (A) MRI of patients without necrosis showed hollow spike-like images without a significant “double line”. (B) Necrosis patients showed a clear “double line” sign and displayed high signals on fat-suppressed images. (C) X-ray of patients without necrosis showed a smooth surface without collapse. (D) Necrosis patients showed femoral head collapse, density changes, cystic changes, or hollow or pierced femoral head surface.

Clinical data of 20 patients are shown in Table 2. There were no significant differences for the two groups of patients in age, blood pressure and other vital signs; Harris scores of the TIONFH group were lower than those without necrosis (p = 0.008), and VAS scores were higher than those without necrosis (p = 0.009). These results indicated that hip function in the necrosis group was significantly limited. MRI test results are shown as follows. In patients without necrosis, there appeared hollow spike-like images without a significant “double line” sign (Fig 1 A). Necrosis patients had a clear “double line” sign and displayed high signals on fat-suppressed images, which is a typical feature of TIONFH (Fig 1 B). X-ray analysis showed that patients without necrosis had a smooth surface without collapse and necrosis patients showed femoral head collapse or density changes, cystic changes, or a hollow or pierced femoral head surface, (Fig 1C and 1D). These findings are typical, demonstrating that the bone structure of TIONFH patients showed significant changes.

**Table 2:** Basic information for a TIONFH patient and patient without necrosis.

groups	Necrosis group(n = 10)	Non-necrosis group(n = 10)
Median age,range(years)	43(20–55)	45.745(36–57)
Sex(male/femal)	5/5	9/1
BMI(g)	24.08±3.31	23.97±3.65
Pulse(time/min)	81.70±8.51	76.90±2.88
Breathe(time/min)	19.10±1.20	19.10±1.45
Body temperature(℃)	36.57±0.31	36.64±0.39
Systolic blood pressure(mmHg)	121.00±5.44	124.10±9.39
diastolic blood pressure(mmHg)	78.10±6.81	78.90±6.38
Harris scores	78.70±13.77	93.10±5.40
VAS scores	1.70±1.49	0.30±0.48

### Histological structure changes of necrotic bone tissue

**Fig 2:**
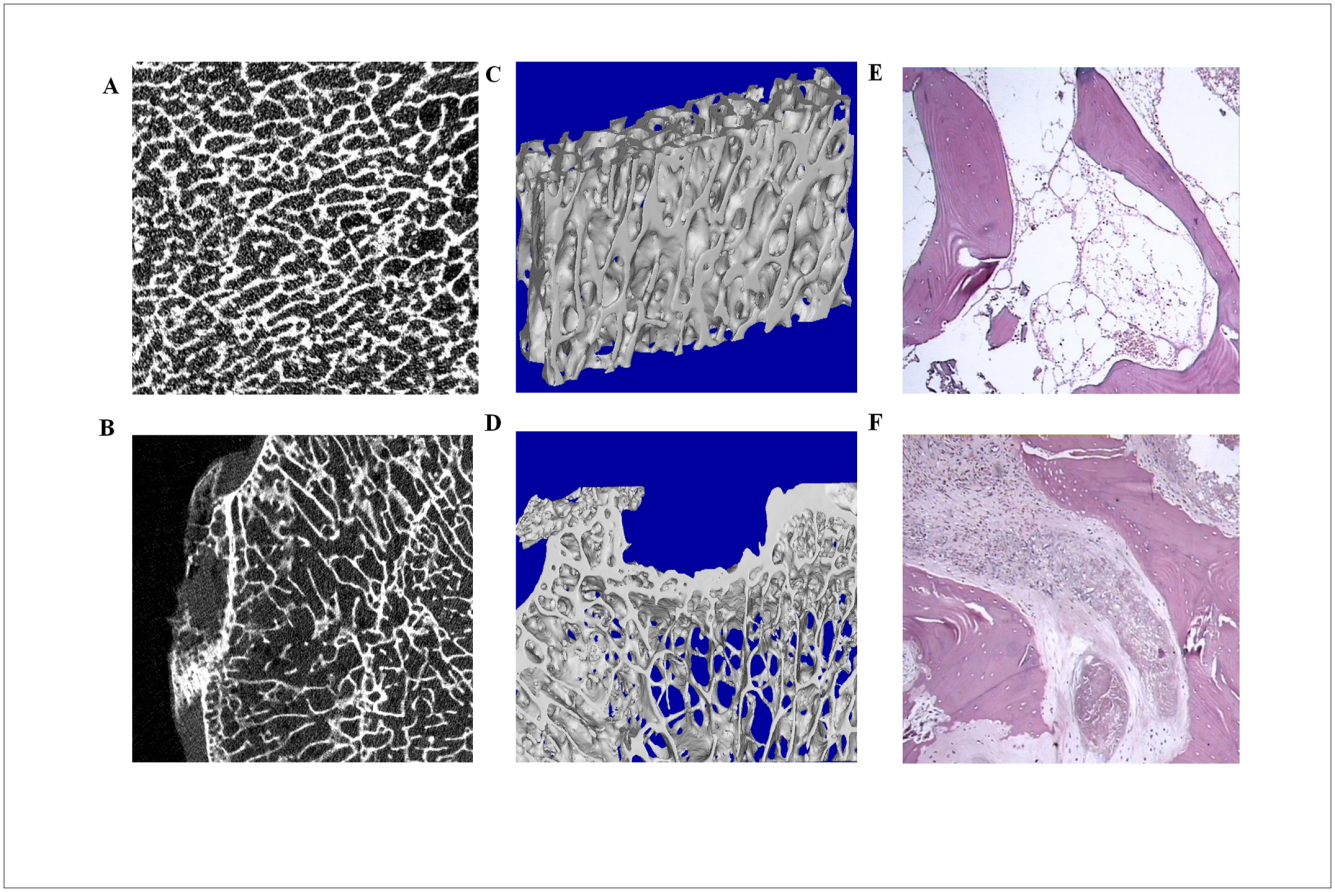
TIONFH patient bone collapse and osteoblast death. (A) Micro-CT of a normal area. (B) Micro-CT of a necrotic area, which appeared to indicate trabecular bone disorder, shows structural relaxation. (C) Three-dimensional reconstruction of normal areas. (D) Three-dimensional reconstruction of a necrotic area showing trabecular bone collapse deformation. (E) HE staining of normal areas. (F) HE staining of a necrotic area shows significant bone necrosis and bone marrow tissue necrosis between the trabecular and simultaneous granulation tissue.

The micro-CT results showed that compared to the normal area, the necrotic area showed structural relaxation (Fig 2A and 2B). Three-dimensional reconstruction showed trabecular bone collapse deformation compared to the normal area (Fig 2C and 2D). Further pathology staining demonstrated a necrotic area with significant bone necrosis and bone marrow tissue necrosis between the trabecular and simultaneous granulation tissue (Fig 2E and 2F). This evidence suggests that TIONFH patients had significant bone structure changes and osteoblast death, which is an important potential cause of bone collapse and hip dysfunction.

### miRNA array of peripheral blood and Q-PCR confirmation

**Fig 3:**
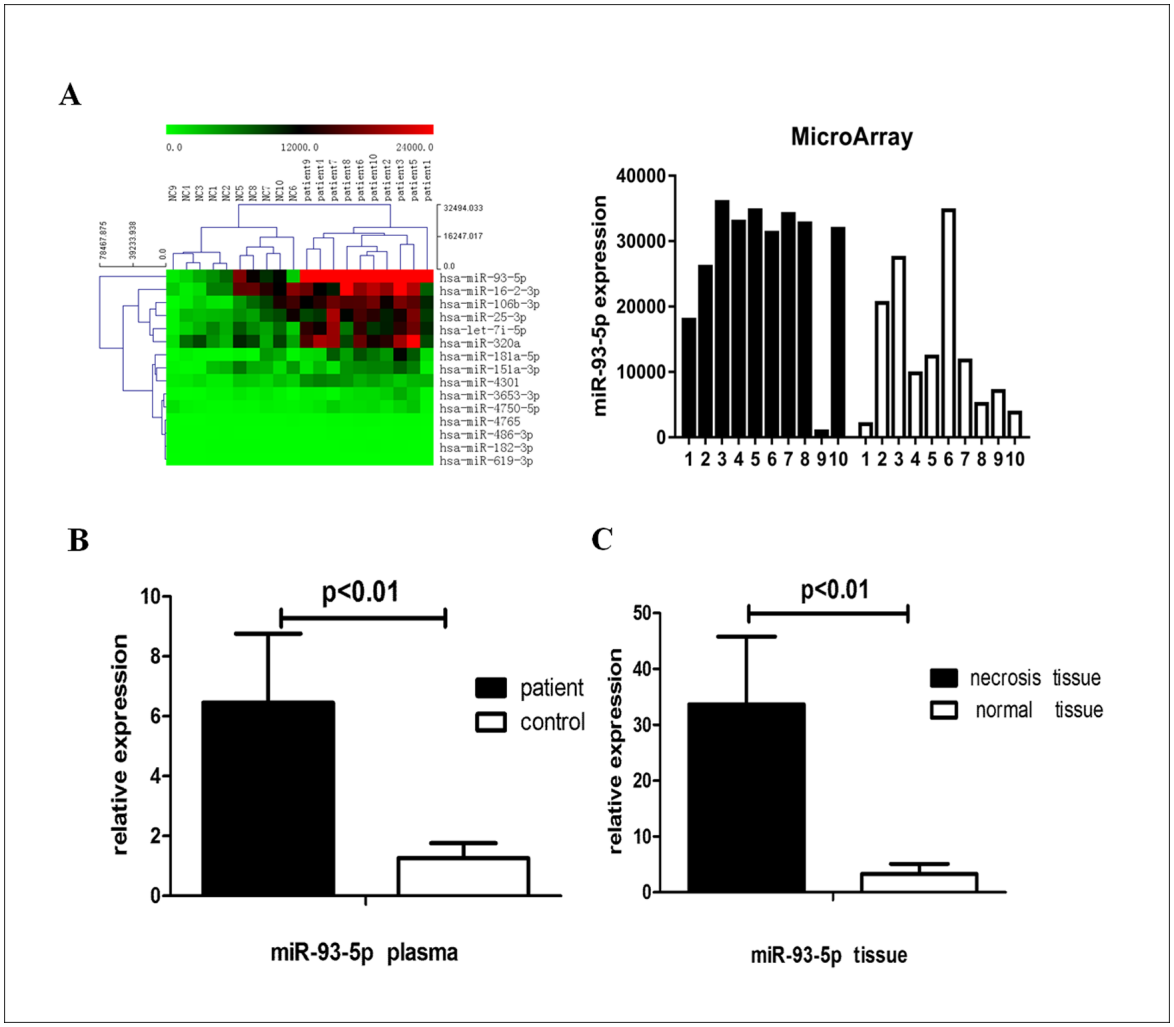
miRNA array and miR-93-5p expression. (A) Cluster analysis of partially differentially expressed miRNAs from TIONFH patients and controls. (B) miR-93-5p expression level in plasma was significantly increased as shown by Q-PCR. (C) miR-93-5p expression increased in bone tissue as shown by Q-PCR.

We analysed TIONFH patients (n = 10) and patients without necrosis after femoral neck fracture (n = 10) by microarray and found 297 differentially expressed miRNAs in peripheral blood. These differences included 35 up-regulated miRNAs, such as miR-93-5p, miR-7i-5p, miR-320a, miR-25-3p, and miR-16-2-3p (fold > 2.0, P < 0.05). A partially differentially expressed miRNA cluster diagram is shown (Fig 3A), and miR-93-5p, with a fold change more than 3, was further examined.

Q-PCR was used to further confirm the miR-93-5p expression and showed that miR-93-5p was significantly increased in the peripheral blood of TIONFH patients (Fig 3B), consistent with the microarray analysis. At the same time, Q-PCR analysis of bone tissue showed that the expression of miR-93-5p in necrotic areas was significantly higher than that in normal areas (Fig 3C).

Whether increased miR-93-5p in TIONFH patients is involved in bone metabolism abnormalities caused by bone necrosis remains to be elucidated. To assess this hypothesis, we performed further experiments.

### Association of miR-93-5p and osteoblast differentiation

**Fig 4:**
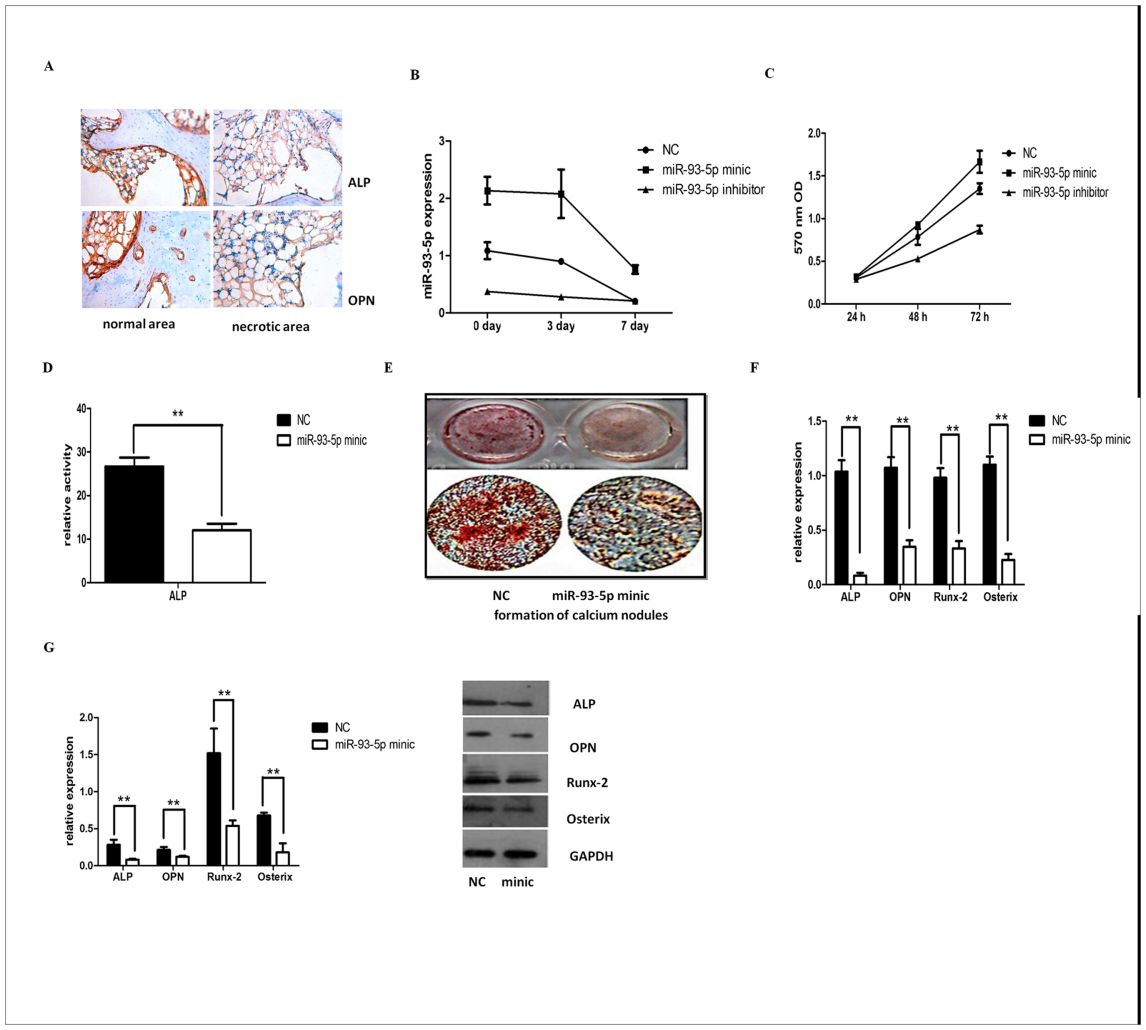
Association of miR-93-5p and osteoblast differentiation. (A) ALP and OPN expression in TIONFH patients with necrotic bone tissue samples by immunohistochemistry. Brown represents positive signals. (B) Real time Q-PCR detected the miR-93-5p expression level during differentiation at 0, 3, and 7 d. (C) Effects of miR-93-5p on hBMSCs] proliferation after 24, 48 and 72 h by MTT assays. (D) ALP enzyme activity was detected with a reagent kit. (E) AR staining to assess mineralisation. Two cell groups were seeded on 96-well plastic dishes (Costar). Cells were cultured in differentiation medium for 21 d, and images were captured using a scanner. (F) mRNA expression level of ALP, OPN, Runx-2 and Osterix using real time PCR analysis. (G) Western blotting was performed to detect protein levels of ALP, OPN, Runx-2 and Osterix. (* P < 0.05 and ** P < 0.01).

Immunohistochemistry of necrotic bone tissue samples from TIONFH patients demonstrated that ALP and OPN expression was decreased (Fig 4A), indicating that patients with TIONFH had a low osteoblast differentiation ability.

Patients with bone necrosis tend to exhibit decreased cell differentiation. Is this process directly related to abnormal expression of miR-93-5p? To address this issue, we further verified the impact of miR-93-5p on osteoblast differentiation in cell experiments.

Cell culture and identification experiments were performed. Flow cytometry revealed that the cultured cells were negative for HLA-DR, CD45, CD34, CD14, and CD11b and positive for CD166, CD105, CD73, CD44, and CD29. These cells are characterised by their surface marker profile [18]; they possessed the stem cell characteristics of hBMSCs and could thus be used for further research.

miR-93-5p expression during hBMSCs differentiation was also assessed. Q-PCR results showed that the miR-93-5p expression was decreased in the NC group when they were cultured in differentiation medium after 3 and 7 d, but the miR-93-5p mimic group showed higher rates of expression compared to the other groups, indicating successful plasmid transfection (Fig 4B)

The results of MTT assays indicated that the miR-93-5p mimic significantly promoted hBMSC cell proliferation in basic medium after 48 h, which was more pronounced after 72 h (p<0.05) (Fig 4C).

To further validate the effect of miR-93-5p on hBMSC osteogenic differentiation, we used an ALP assay kit to test ALP activity of hBMSCs that were induced for 7 d with an osteogenic agent. The results showed that ALP activity in the mimic group was significantly inhibited (Fig 4D). Meanwhile, AR staining of osteogenic agent-induced cells was performed after 21 d. The formation of calcium nodules was significantly reduced in the mimic group. (Fig 4E). Q-PCR and Western blotting were used to detect the gene and protein changes of bone formation-related indicators, including ALP, OPN, Runx-2 and Osterix, after osteogenic agent induction for 7 d. The results showed that the gene and protein levels of ALP, OPN, Runx-2 and Osterix were significantly suppressed in the mimic group (Fig 4F and 4G). These data indicated that miR-93-5p can suppress hBMSC osteogenic differentiation in vitro and suggest the important role of miR-93-5p in osteogenic differentiation.

### Identification of BMP-2 as a miR-29 target

**Fig 5:**
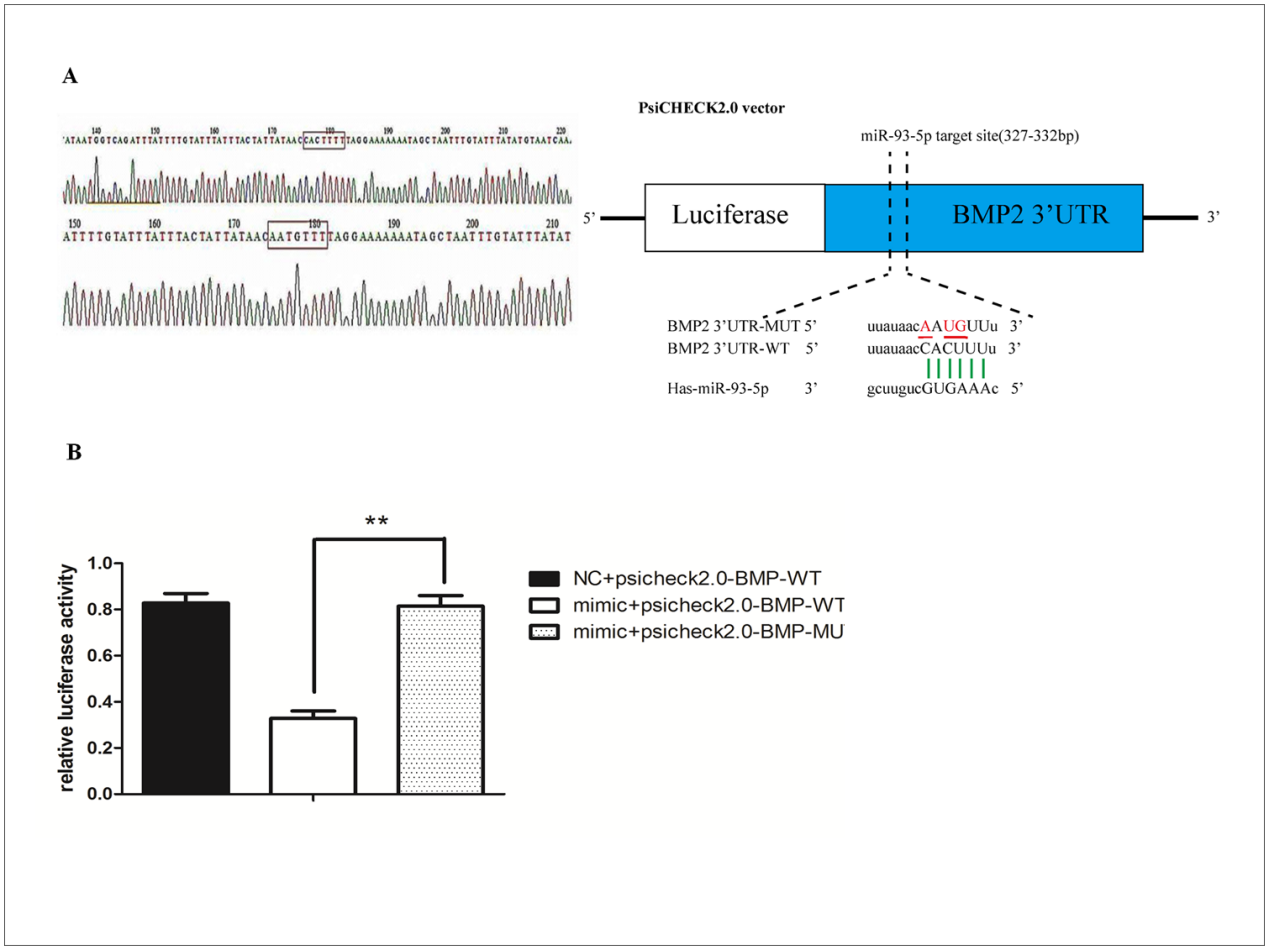
Identification of BMP-2 as a target of miR-93-5p. (A) miR-93-5p targets the 3 'UTR of BMP-2. BMP-2-3'UTR WT or MUT was cloned into psiCHECK2.0 and co-transfected with the miR-93-5p mimic. miR-93-5p mimics bind specifically to BMP2-3'UTR WT. (B) Effect of miR-93-5p on BMP-2 RNA 3'UTR region by the luciferase gene reporter assay. (* P < 0.05 and ** P < 0.01).

Online software was used to screen potential miR-93-5p targets. Based on the complementarity with the miR-93-5p seed sequence, we focused BMP-2 (Fig 5A). The BMP2 3′-UTR with either the predicted WT miR-93-5p binding site or a mutant binding site was inserted into the psiCHECK2 vector (Fig 5A). WT-BMP2 significantly repressed the expression of Renilla luciferase without affecting the MUT-BMP2 (Fig 5B). These results demonstrated that miR-93-5p can bind to the 3′-UTR of the BMP-2.

Luciferase gene reporter experiments showed that in the WT group, luciferase activity was significantly reduced due to binding to the miR-93-5p mimic, while the miR-93-5p mimic did not affect the luciferase activity of the MUT group (Fig 5C). These results demonstrate that miR-93-5p inhibits the expression of BMP-2 by binding to a specific region of the BMP-2 miRNA 3'UTR.

### miR-93-5p regulation of BMP-2 expression

**Fig 6:**
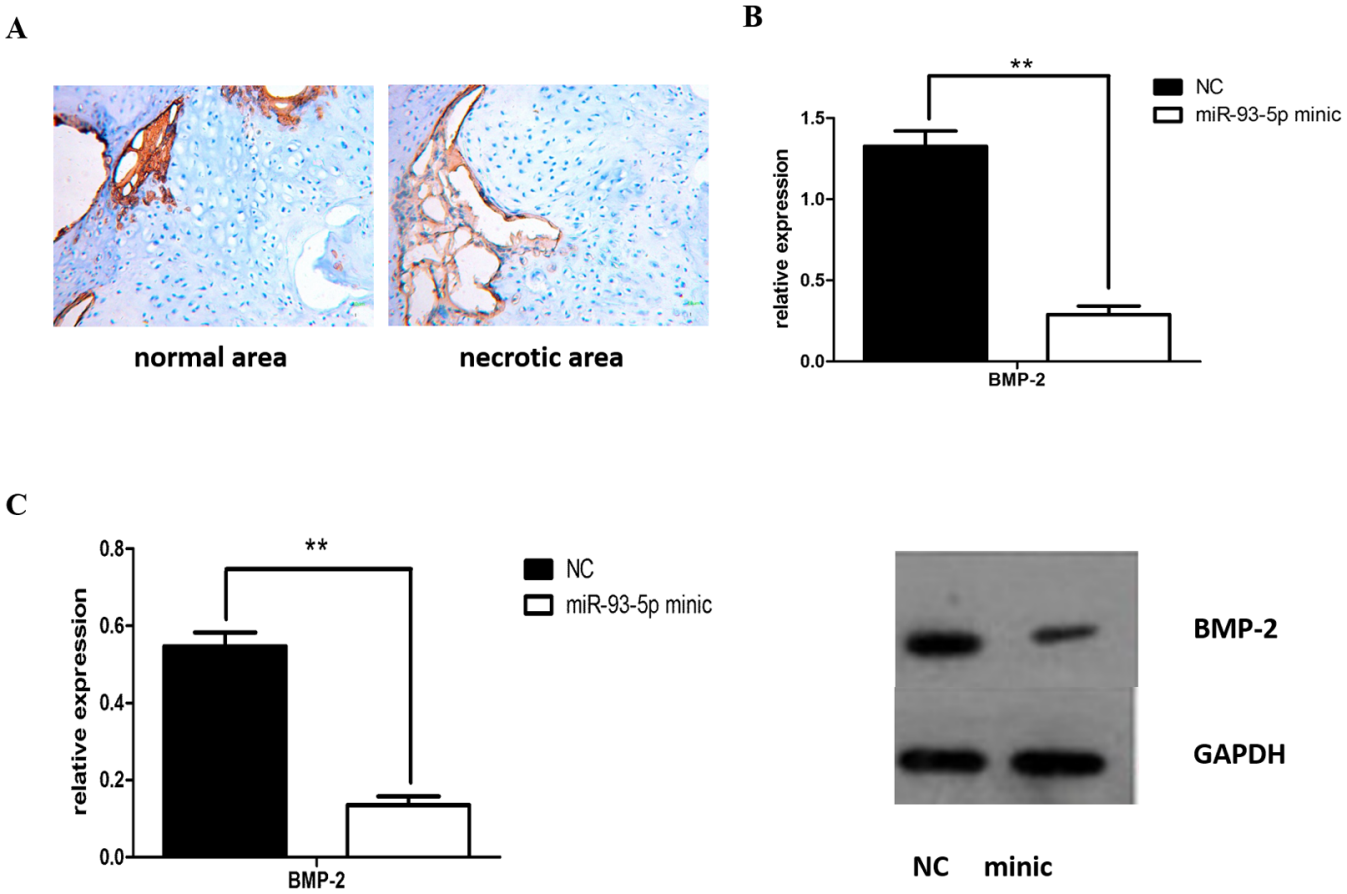
miR-93-5p regulation of BMP-2 expression. (A) BMP-2 immunohistochemical analysis of normal bone tissue from patients with necrotic areas and the area of necrosis. (B) BMP-2 protein expression in hBMSCs by Western blotting analysis. (C) BMP-2 mRNA expression level in hBMSCs by real time Q-PCR analysis. (* P < 0.05 and ** P < 0.01).

Immunohistochemical analysis of samples showed that BMP-2 was significantly reduced, as shown in Fig 6A. In vivo, we further validated the gene and protein levels of BMP-2. BMP-2 expression was significantly reduced in the Inducer + miR-93-5p mimic group (Fig 6B and 6C), which is consistent with the luciferase reporter gene results. The above results confirmed that low expression of the osteogenic protein BMP-2 was associated with high miR-93-5p expression in TIONFH patients and decreased osteoblast differentiation.

## Discussion

This study reveals a major mechanism of TIONFH patient bone collapse. TIONFH is a femoral head blood circulation disorder with various causes that can result in in bone cell and bone marrow component death and then structural changes in the femoral head. Early necrosis occurs in the outer range of the femoral head and is characterised by increased local bone mineral density with unclear trabecular bone and a flat area showing collapse in the avascular necrosis area. Avoiding femoral head necrosis and facilitating fracture healing is pivotal for treatment of this condition. Therefore, exploring osteoblast differentiation and proliferation as well as promoting osteogenic potential of the femoral head in patients is crucial for the prevention and treatment of this condition. The BMP-2 gene has been genetically linked to osteoporosis and osteoarthritis [19]. Abnormal BMP-2 levels cause congenital anomalies and diseases involving the mesenchymal cells, which differentiate into muscle, fat, cartilage, and bone [20]. The 3'UTR of BMP-2 genes from mammals to fish are extraordinarily conserved, which indicates that the BMP-2 3'UTR is under stringent selective pressure, and the conserved region is a strong posttranscriptional regulator of BMP-2 expression [19]. In this study, we demonstrated that miR-93-5p posttranscriptionally regulates BMP-2 by binding to the 3'UTR. Our study provides the first evidence that TIONFH patients have high miR-93-5p expression in the peripheral blood and that TIONFH inhibits osteoblast differentiation by reducing BMP-2 expression.

miR-93-5p, as a member of the microRNA gene cluster miR-106b~25 (miR-106b, miR-93, and miR-25), is expressed in primary stem cells [21] and normal tissue [22] as well as in pathological contexts, such as tumour development [23], ageing [24], bone formation [25], and cardiovascular disease [26]. This miRNA promotes cell proliferation in osteosarcoma [27], bladder cancer [28], and neural stem cells [29]. However, miR-93-5p was also shown to inhibit cell expansion in human colon cancer [30]. The coexistence of these seemingly contradictory effects suggests that the function of this miRNA is related to the specific tissue or microenvironment involved [31].

In recent years, the roles of small RNAs in orthopaedic diseases have been investigated and analysed. miRNAs are important for osteogenic differentiation. miR-204 and miR140-5p were shown to regulate the proliferation of BMSCs, and miR140-5p was associated with the expression of BMP-2 [2,32]. However, miRNAs involved in TIONFH have not been unidentified. We screening genes between TIONFH patients and patients without necrosis and demonstrated that miR-93-5p was increased in the subjects, which was verified by Q-PCR. These results provide potential new diagnostic strategies for TIONFH.

Notably, while screening miRNA, we chose postoperative necrosis patients after trauma without necrosis rather than healthy individuals as controls. Therefore, interference from hormonal necrosis or other factors were ruled out.

To further explore the mechanism of miR-93-5p in TIONFH, we analysed its target gene, BMP-2, after prediction by bioinformatics methods and luciferase reporter gene analysis. In addition, in situ expression of BMP-2 in TIONFH patients was tested by immunochemistry. BMP-2 is an important protein in osteogenic differentiation, and miR-93-5p is a negative regulator of BMP-2. miR-93-5p and the BMP-2 RNA 3'UTR region binding were not completely paired, and thus, the function of hBMSCs expressing BMP-2 was inhibited. This blocks bone-related transcription of other genes in the cells, suppressing the formation of osteoblasts. In vivo, BMP-2 protein expression was low in tissues of femoral head necrosis patients and had an inverse correlation with the miR-93-5p level. This is consistent with the in vitro results. Therefore, miR-93-5p likely inhibits osteoblast differentiation via BMP-2. These observations contribute to our understanding of BMP-2-associated miRNAs and also elucidate the mechanism of TIONFH-mediated inhibition of osteogenic differentiation.

ALP is an important indicator of early differentiation in osteogenic differentiation. OPN is involved in osteoblast adhesion and mineralisation [33]. In patient tissue samples, we found that ALP and OPN were significantly reduced. Furthermore, we performed in vitro studies. The regulation of miRNAs was time-dependent and based on the time required for osteoblast differentiation and mineralisation (7 d and 21 d, respectively). At the corresponding time, the formation of ALP and calcium nodules and the expression of osteogenic genes in hBMSCs all decreased. In vitro and in vivo experiments indicated that osteogenic differentiation inhibition was associated with miR-93-5p. Combined with clinical results showing that BMP-2 was decreased, these findings indicated that TIONFH bone lesions occur in patients with hip collapse phenomenon. The level of Runx-2 determines the degree of skeletal maturation and the rate of conversion [34], and Osterix is expressed in all developing osteocytes and osteoblasts; these proteins are downstream molecules of BMP-2 signalling [35,36,37]. Thus, based on multiple lines of evidence, miR-93-5p inhibits osteogenic differentiation by BMP-2.

Interestingly, we found that miR-93-5p promoted cell proliferation of hBMSCs during in vitro experiments. Other evidence, such as the significantly reduced ALP and the formation of calcium nodules as well as the expression of BMP-2, Runx-2 and Osterix, demonstrated the inhibition of bone cell differentiation in response to miR-93-5p. However, this is a complex and interesting issue. These results prompted us to assess whether hBMSCs have multi-lineage differentiation potential [38] and whether miR-93-5p induces hBMSCs toward other types of differentiation. These questions should be further investigated and will promote enhanced understanding and novel treatment approaches. Currently, although the function of miR-93-5p in promoting hBMSC proliferation and suppressing osteogenic differentiation is unclear, specific suppression of miR-93-5p in hBMSC osteogenic differentiation has been demonstrated.

Based on comprehensive experimental results in vitro and in vivo, miR-93-5p was shown to be significantly increased in TIONFH patients, and its osteogenic suppression function was related to target genes, such as BMP-2, which may be one of the mechanisms of bone necrosis collapse. Our findings improve our understanding of the osteonecrosis mechanisms. However, increased miR-93-5p will have broad pleiotropic effects. As with all miRNAs, miR-93-5p has many targets, and BMP-2 is just one target. It is unclear whether the effects of miR-93-5p on osteogenic differentiation depend solely/primarily on targeting BMP-2 or whether other targets are involved as well. In this study, we only assessed the effects of miR-93-5p in TIONFH, and more detailed examinations are required. These studies provide a new approach for diagnosing and treating TIONFH as well as other diseases associated with bone necrosis, such as steroid femoral head necrosis.

## Supporting information

S1 Figs Original uncropped and unadjusted Western blotting of ALP, OPN, Runx-2, Osterix, BMP-2, GAPDH. (RAR)

S1 File Editorial certification from AJE. (PDF)

S1 Data Microarray data on GEO. The related data were submitted to GEO as required, and the following link has been created to allow review of record GSE89587 while it remains in private status: https://www.ncbi.nlm.nih.gov/geo/query/acc.cgi?token=clefscoqbpgxdid&;acc=GSE89587
(RAR)

## References

[1] Xu JF, Yang GH, Pan XH, Zhang SJ, Zhao C, Qiu BS, et al Altered microRNA expression profile in exosomes during osteogenic differentiation of human bone marrow-derived mesenchymal stem cells. PLoS One. 2014; 9: e114627 doi: 10.1371/journal.pone.0114627 25503309 10.1371/journal.pone.0114627 PMC4263734

[2] Hwang S, Park SK, Lee HY, Kim SW, Lee JS, Choi EK, et al miR-140-5p suppresses BMP2-mediated osteogenesis in undifferentiated human mesenchymal stem cells. FEBS Lett. 2014; 588: 2957–2963. doi: 10.1016/j.febslet.2014.05.048 24928442 10.1016/j.febslet.2014.05.048

[3] Maes OC, An J, Sarojini H, Wang E. Murine microRNAs implicated in liver functions and aging process. Mech Ageing Dev. 2008; 129: 534–541. doi: 10.1016/j.mad.2008.05.004 18561983 10.1016/j.mad.2008.05.004

[4] Heo JS, Choi Y, Kim HS, Kim HO. Comparison of molecular profiles of human mesenchymal stem cells derived from bone marrow, umbilical cord blood, placenta and adipose tissue. Int J Mol Med. 2016; 37: 115–125. doi: 10.3892/ijmm.2015.2413 26719857 10.3892/ijmm.2015.2413 PMC4687432

[5] Brocher J, Janicki P, Voltz P, Seebach E, Neumann E, Mueller-Ladner U, et al Inferior ectopic bone formation of mesenchymal stromal cells from adipose tissue compared to bone marrow: rescue by chondrogenic pre-induction. Stem Cell Res. 2013; 11: 1393–1406. doi: 10.1016/j.scr.2013.07.008 24140198 10.1016/j.scr.2013.07.008

[6] Tsuji K, Bandyopadhyay A, Harfe BD, Cox K, Kakar S, Gerstenfeld L, et al BMP2 activity, although dispensable for bone formation, is required for the initiation of fracture healing. Nat Genet. 2006; 38: 1424–1429. doi: 10.1038/ng1916 17099713 10.1038/ng1916

[7] Wang L, Park P, La Marca F, Than K, Rahman S, Lin CY. Bone formation induced by BMP-2 in human osteosarcoma cells. Int J Oncol. 2013; 43: 1095–1102. doi: 10.3892/ijo.2013.2030 23900689 10.3892/ijo.2013.2030 PMC3829777

[8] Dai J, Li Y, Zhou H, Chen J, Chen M, Xiao Z. Genistein promotion of osteogenic differentiation through BMP2/SMAD5/RUNX2 signaling. Int J Biol Sci. 2013; 9: 1089–1098. doi: 10.7150/ijbs.7367 24339730 10.7150/ijbs.7367 PMC3858582

[9] Shu B, Zhang M, Xie R, Wang M, Jin H, Hou W, et al BMP2, but not BMP4, is crucial for chondrocyte proliferation and maturation during endochondral bone development. J Cell Sci. 2011; 124: 3428–3440. doi: 10.1242/jcs.083659 21984813 10.1242/jcs.083659 PMC3196857

[10] Bandyopadhyay A, Tsuji K, Cox K, Harfe BD, Rosen V, Tabin CJ. Genetic analysis of the roles of BMP2, BMP4, and BMP7 in limb patterning and skeletogenesis. PLoS Genet. 2006; 2: e216 doi: 10.1371/journal.pgen.0020216 17194222 10.1371/journal.pgen.0020216 PMC1713256

[11] Zhao DW, Hu YC. Adult femoral head necrosis diagnosis and treatment standards expert consensus (2012 edition). Chinese Journal of Joint Surgery (Electronic Edition). 2012; 479–484.

[12] Mahomed NN, Arndt DC, McGrory BJ, Harris WH. The Harris hip score: comparison of patient self-report with surgeon assessment. J Arthroplasty. 2001; 16: 575–580. 11503116 10.1054/arth.2001.23716

[13] Mantha S, Thisted R, Foss J, Ellis JE, Roizen MF. A proposal to use confidence intervals for visual analog scale data for pain measurement to determine clinical significance. Anesth Analg. 1993; 77: 1041–1047. 8214704 10.1213/00000539-199311000-00029

[14] Shichinohe H, Kuroda S, Sugiyama T, Ito M, Kawabori M, Nishio M, et al Biological Features of Human Bone Marrow Stromal Cells (hBMSC) Cultured with Animal Protein-Free Medium-Safety and Efficacy of Clinical Use for Neurotransplantation. Transl Stroke Res. 2011; 2: 307–315. doi: 10.1007/s12975-011-0088-y 24323651 10.1007/s12975-011-0088-y

[15] Larsen KH, Frederiksen CM, Burns JS, Abdallah BM, Kassem M. Identifying a molecular phenotype for bone marrow stromal cells with in vivo bone-forming capacity. J Bone Miner Res. 2010; 25: 796–808. doi: 10.1359/jbmr.091018 19821776 10.1359/jbmr.091018

[16] Duttenhoefer F, Hieber SF, Stricker A, Schmelzeisen R, Gutwald R, Sauerbier S. Follow-up of implant survival comparing ficoll and bone marrow aspirate concentrate methods for hard tissue regeneration with mesenchymal stem cells in humans. Biores Open Access. 2014; 3: 75–76. doi: 10.1089/biores.2014.0003 24804168 10.1089/biores.2014.0003 PMC3995205

[17] Zhu H, Miosge N, Schulz J, Schliephake H. Regulation of multilineage gene expression and apoptosis during in vitro expansion of human bone marrow stromal cells with different cell culture media. Cells Tissues Organs. 2010; 192: 211–220. doi: 10.1159/000313417 20407225 10.1159/000313417

[18] Kamath MS, Ahmed SS, Dhanasekaran M, Santosh SW. Polycaprolactone scaffold engineered for sustained release of resveratrol: therapeutic enhancement in bone tissue engineering. Int J Nanomedicine. 2014; 9: 183–195. 10.2147/IJN.S49460 PMC3875521 24399875

[19] Rogers MB, Shah TA, Shaikh NN. Turning Bone Morphogenetic Protein 2 (BMP2) on and off in Mesenchymal Cells. J Cell Biochem. 2015; 116: 2127–2138. doi: 10.1002/jcb.25164 25776852 10.1002/jcb.25164 PMC4862620

[20] Fritz DT, Jiang S, Xu J, Rogers MB. A polymorphism in a conserved posttranscriptional regulatory motif alters bone morphogenetic protein 2 (BMP2) RNA:protein interactions. Mol Endocrinol. 2006; 20: 1574–1586. doi: 10.1210/me.2005-0469 16497730 10.1210/me.2005-0469

[21] Brett JO, Renault VM, Rafalski VA, Webb AE, Brunet A. The microRNA cluster miR-106b~25 regulates adult neural stem/progenitor cell proliferation and neuronal differentiation. Aging (Albany NY). 2011; 3: 108–124. 21386132 10.18632/aging.100285 PMC3082007

[22] Tang Q, Zou Z, Zou C, Zhang Q, Huang R, Guan X, et al MicroRNA-93 suppress colorectal cancer development via Wnt/beta-catenin pathway downregulating. Tumour Biol. 2015; 36: 1701–1710. doi: 10.1007/s13277-014-2771-6 25371073 10.1007/s13277-014-2771-6

[23] Fang L, Deng Z, Shatseva T, Yang J, Peng C, Du WW, et al MicroRNA miR-93 promotes tumor growth and angiogenesis by targeting integrin-beta8. Oncogene. 2011; 30: 806–821. doi: 10.1038/onc.2010.465 20956944 10.1038/onc.2010.465

[24] Li N, Muthusamy S, Liang R, Sarojini H, Wang E. Increased expression of miR-34a and miR-93 in rat liver during aging, and their impact on the expression of Mgst1 and Sirt1. Mech Ageing Dev. 2011; 132: 75–85. doi: 10.1016/j.mad.2010.12.004 21216258 10.1016/j.mad.2010.12.004

[25] Hazarika S, Farber CR, Dokun AO, Pitsillides AN, Wang T, Lye RJ, et al MicroRNA-93 controls perfusion recovery after hindlimb ischemia by modulating expression of multiple genes in the cell cycle pathway. Circulation. 2013; 127: 1818–1828. doi: 10.1161/CIRCULATIONAHA.112.000860 23559675 10.1161/CIRCULATIONAHA.112.000860 PMC3856290

[26] Dickinson BA, Semus HM, Montgomery RL, Stack C, Latimer PA, Lewton SM, et al Plasma microRNAs serve as biomarkers of therapeutic efficacy and disease progression in hypertension-induced heart failure. Eur J Heart Fail. 2013; 15: 650–659. doi: 10.1093/eurjhf/hft018 23388090 10.1093/eurjhf/hft018

[27] Kawano M, Tanaka K, Itonaga I, Ikeda S, Iwasaki T, Tsumura H. microRNA-93 promotes cell proliferation via targeting of PTEN in Osteosarcoma cells. J Exp Clin Cancer Res. 2015; 34: 76 doi: 10.1186/s13046-015-0192-z 26243299 10.1186/s13046-015-0192-z PMC4524362

[28] Armstrong DA, Green BB, Seigne JD, Schned AR, Marsit CJ. MicroRNA molecular profiling from matched tumor and bio-fluids in bladder cancer. Mol Cancer. 2015; 14: 194 doi: 10.1186/s12943-015-0466-2 26576778 10.1186/s12943-015-0466-2 PMC4650939

[29] Liu H, Han XH, Chen H, Zheng CX, Yang Y, Huang XL. Repetitive magnetic stimulation promotes neural stem cells proliferation by upregulating MiR-106b in vitro. J Huazhong Univ Sci Technolog Med Sci. 2015; 35: 766–772. doi: 10.1007/s11596-015-1505-3 26489637 10.1007/s11596-015-1505-3

[30] Xiao ZG, Deng ZS, Zhang YD, Zhang Y, Huang ZC. Clinical significance of microRNA-93 downregulation in human colon cancer. Eur J Gastroenterol Hepatol. 2013; 25: 296–301. doi: 10.1097/MEG.0b013e32835c077a 23354160 10.1097/MEG.0b013e32835c077a

[31] Liu S, Zhang P, Chen Z, Liu M, Li X, Tang H. MicroRNA-7 downregulates XIAP expression to suppress cell growth and promote apoptosis in cervical cancer cells. FEBS Lett. 2013; 587: 2247–2253. doi: 10.1016/j.febslet.2013.05.054 23742934 10.1016/j.febslet.2013.05.054

[32] Huang J, Zhao L, Xing L, Chen D. MicroRNA-204 regulates Runx2 protein expression and mesenchymal progenitor cell differentiation. Stem Cells. 2010; 28: 357–364. doi: 10.1002/stem.288 20039258 10.1002/stem.288 PMC2837600

[33] Noda M, Tsuji K, Nifuji A. [Osteopontin: a topic from the point of bone morphology]. Clin Calcium. 2003; 13: 464–466. 15775113

[34] Maruyama Z, Yoshida CA, Furuichi T, Amizuka N, Ito M, Fukuyama R, et al Runx2 determines bone maturity and turnover rate in postnatal bone development and is involved in bone loss in estrogen deficiency. Dev Dyn. 2007; 236: 1876–1890. doi: 10.1002/dvdy.21187 17497678 10.1002/dvdy.21187

[35] Jang H, Kim EJ, Park JK, Kim DE, Kim HJ, Sun WS, et al SMILE inhibits BMP-2-induced expression of osteocalcin by suppressing the activity of the RUNX2 transcription factor in MC3T3E1 cells. Bone. 2014; 61: 10–18. doi: 10.1016/j.bone.2013.12.028 24389415 10.1016/j.bone.2013.12.028

[36] Ming LG, Chen KM. Two important signal transduction pathways in bone formation. Chinese Journal of Osteoporosis. 2011; 275–278.

[37] Jeon EJ, Lee KY, Choi NS, Lee MH, Kim HN, Jin YH, et al Bone morphogenetic protein-2 stimulates Runx2 acetylation. J Biol Chem. 2006; 281: 16502–16511. doi: 10.1074/jbc.M512494200 16613856 10.1074/jbc.M512494200

[38] Baksh D, Yao R, Tuan RS. Comparison of proliferative and multilineage differentiation potential of human mesenchymal stem cells derived from umbilical cord and bone marrow. Stem Cells. 2007; 25: 1384–1392. doi: 10.1634/stemcells.2006-0709 17332507 10.1634/stemcells.2006-0709

